# Phosphorylation of mixed lineage kinase MLK3 by cyclin-dependent kinases CDK1 and CDK2 controls ovarian cancer cell division

**DOI:** 10.1016/j.jbc.2022.102263

**Published:** 2022-07-14

**Authors:** Luis Cedeno-Rosario, David Honda, Autumn M. Sunderland, Mark D. Lewandowski, William R. Taylor, Deborah N. Chadee

**Affiliations:** Department of Biological Sciences, College of Natural Sciences and Mathematics, The University of Toledo, Toledo, Ohio, USA

**Keywords:** mixed lineage kinase 3, c-Jun N-terminal kinase, cyclin-dependent kinase, cell division, cell proliferation, cell signaling, ovarian cancer, B-Raf, v-Raf murine sarcoma viral oncogene homolog B1, BrdU, bromodeoxyuridine, BSA, bovine serum albumin, CDK1, cyclin-dependent kinase 1, CIAP, calf intestinal phosphatase, CPC, chromosomal passenger complex, CRIB, Cdc42/Rac interactive binding, DAPI, 4′,6-diamidino-2-phenylindole, DMSO, dimethyl sulfoxide, DTB, double thymidine block, ERK, extracellular signal–regulated kinase, GST, glutathione-*S*-transferase, HEK293, human embryonic kidney 293 cell line, JNK, c-Jun N-terminal kinase, KD, kinase dead, MAPK, mitogen-activated protein kinase, MAP3K, mitogen-activated protein kinase kinase kinase, MLK3, mixed lineage kinase 3, p-CDK1, phospho-CDK1, p-CDK2, phospho-CDK2, p-Histone H3, phospho-Histone H3, p-JNK, phospho-JNK, p-MLK3, phospho-MLK3, p-Rb, phospho-Rb, p-SEK1, phospho-SEK1, Rb, retinoblastoma protein

## Abstract

Mixed lineage kinase 3 (MLK3) is a serine/threonine mitogen-activated protein kinase kinase kinase that promotes the activation of multiple mitogen-activated protein kinase pathways and is required for invasion and proliferation of ovarian cancer cells. Inhibition of MLK activity causes G2/M arrest in HeLa cells; however, the regulation of MLK3 during ovarian cancer cell cycle progression is not known. Here, we found that MLK3 is phosphorylated in mitosis and that inhibition of cyclin-dependent kinase 1 (CDK1) prevented MLK3 phosphorylation. In addition, we observed that c-Jun N-terminal kinase, a downstream target of MLK3 and a direct target of MKK4 (SEK1), was activated in G2 phase when CDK2 activity is increased and then inactivated at the beginning of mitosis concurrent with the increase in CDK1 and MLK3 phosphorylation. Using *in vitro* kinase assays and phosphomutants, we determined that CDK1 phosphorylates MLK3 on Ser548 and decreases MLK3 activity during mitosis, whereas CDK2 phosphorylates MLK3 on Ser770 and increases MLK3 activity during G1/S and G2 phases. We also found that MLK3 inhibition causes a reduction in cell proliferation and a cell cycle arrest in ovarian cancer cells, suggesting that MLK3 is required for ovarian cancer cell cycle progression. Taken together, our results suggest that phosphorylation of MLK3 by CDK1 and CDK2 is important for the regulation of MLK3 and c-Jun N-terminal kinase activities during G1/S, G2, and M phases in ovarian cancer cell division.

Mixed lineage kinase 3 (MLK3) is a serine/threonine mitogen-activated protein kinase (MAPK) kinase kinase (MAP3K) (1, 2, 3) that is required for invasion and proliferation of ovarian cancer cells, and its activity and expression are higher in ovarian cancer cells than normal ovarian epithelial cells (4, 5, 6). In addition to ovarian cancer, MLK3 also plays an essential role in controlling cell proliferation, migration, invasion, and metastasis in other type of cancers, including triple-negative breast cancer, gastric cancer, liver cancer, colorectal cancer, and melanoma (6, 7, 8, 9, 10, 11, 12, 13, 14, 15). In breast cancer cells, MLK3 regulates invasion and migration through c-Jun N-terminal kinase (JNK) signaling and paxillin phosphorylation to drive metastasis (7, 8). Furthermore, MLK3 regulates matrix metalloproteinases, which are required for ovarian and breast cancer cell invasion (4, 14). Moreover, MLK3 expression is targeted by miRNAs to promote melanoma proliferation and invasion as well as liver cancer cell migration (12, 13).

MLK3 is a member of the MLK subfamily of protein kinases, which phosphorylate and activate the MAPK kinase MKK4 (SEK1)/MKK7 and MKK3/6, which in turn phosphorylate and activate the MAPKs JNK and p38 (2, 3, 16, 17, 18, 19, 20). MLK3 can also activate the extracellular signal–regulated kinase (ERK) pathway directly through phosphorylation of MEK and indirectly through regulation of B-Raf (v-Raf murine sarcoma viral oncogene homolog B1) (21, 22, 23). The MLK3 protein contains an SH3 domain, a kinase domain, a leucine-zipper domain, a proline-rich region, and a Cdc42/Rac interactive binding (CRIB) domain (16, 24). Within the kinase domain, there are two autophosphorylation sites that are required for MLK3 enzymatic activity, Thr277 and Ser281 (25). Moreover, the leucine-zipper domain promotes MLK3 homodimerization, which is essential for its activation of the JNK pathway (26). MLK3 kinase activity is autoinhibited by an interaction between the MLK3 SH3 domain and a proline residue located between the leucine-zipper and CRIB domains. This autoinhibition is disrupted when active Cdc42/Rac binds to the CRIB domain (27, 28, 29). MLK3 kinase activity is also increased in response to tumor necrosis factor alpha and interleukin-1β stimulation in ovarian cancer cells (30, 31), and through its phosphorylation by MAP4K4 (Thr738) in pancreatic cancer cells (32). Moreover, activation of the B-Raf–MEK–ERK pathway by oxidative stress creates a positive feedback loop in which reactive oxygen species promotes ERK-dependent phosphorylation of MLK3 on Ser705 and Ser758, which is required for reactive oxygen species–induced activation of ERK and invasion in colon cancer cells (15).

Cyclin-dependent kinases (CDKs)/cyclins and the chromosomal passenger complex (CPC) are master regulators of cell cycle progression and mitotic exit/cytokinesis, respectively (33, 34). During the G2/M transition, CDK1/cyclin B is activated by Cdc25 phosphatase, which promotes the transition into mitosis (35, 36, 37, 38, 39, 40, 41). The CPC regulates several processes during mitosis, including chromosome alignment, histone modification, and cytokinesis (42). The dynamic localization of the CPC during mitosis is mediated by multiple interactions between Borealin, Survivin, INCENP with histones, DNA, and microtubules. Moreover, Aurora B bound to INCENP phosphorylates key proteins to modulate chromosome alignment and cytokinesis (42). The MAPK JNK regulates mitotic progression through phosphorylation of Aurora B and Histone H3 (43, 44). In addition, JNK regulates cell cycle entry and the G2/M DNA damage checkpoint by phosphorylation of Cdc25C on Ser168 and inhibition of CDK1 activity (45, 46, 47).

MLK3 is required for proliferation of ovarian cancer cells (6); however, very little is known about how MLK3 kinase activity is regulated during the cell cycle. In HeLa cells, inhibition of MLK activity resulted in G2/M arrest (48). Moreover, inhibition of MLK activity also causes G2/M arrest and apoptosis in estrogen receptor–positive breast cancer cells (49). Our current findings demonstrate that MLK3 is phosphorylated in late G2 phase by CDK1, and this phosphorylation inhibits MLK3 kinase activity. Furthermore, in synchronized SKOV3 ovarian cancer cells, JNK activity increases through G1/S and G2 phases and is decreased in late G2 concurrent with MLK3 phosphorylation. Our results from *in vitro* kinase assays demonstrate that CDK1 phosphorylates MLK3 on Ser548 during G2/M phase, and CDK2 phosphorylates MLK3 on Ser770 in late G1/S and G2 phases. In addition, inhibition of MLK3 activity with the MLK3 inhibitors URMC099 and CEP1347 resulted in a decrease in cell proliferation and a cell cycle arrest in SKOV3, TOV112D, and HEY ovarian cancer cells. Taken together, our findings suggest that CDK1 and CDK2 regulate the activation of MLK3 and JNK during G1/S and G2/M phases to control ovarian cancer cell division.

## Results

### MLK3 is phosphorylated in mitotic ovarian cancer cells

**Figure 1:**
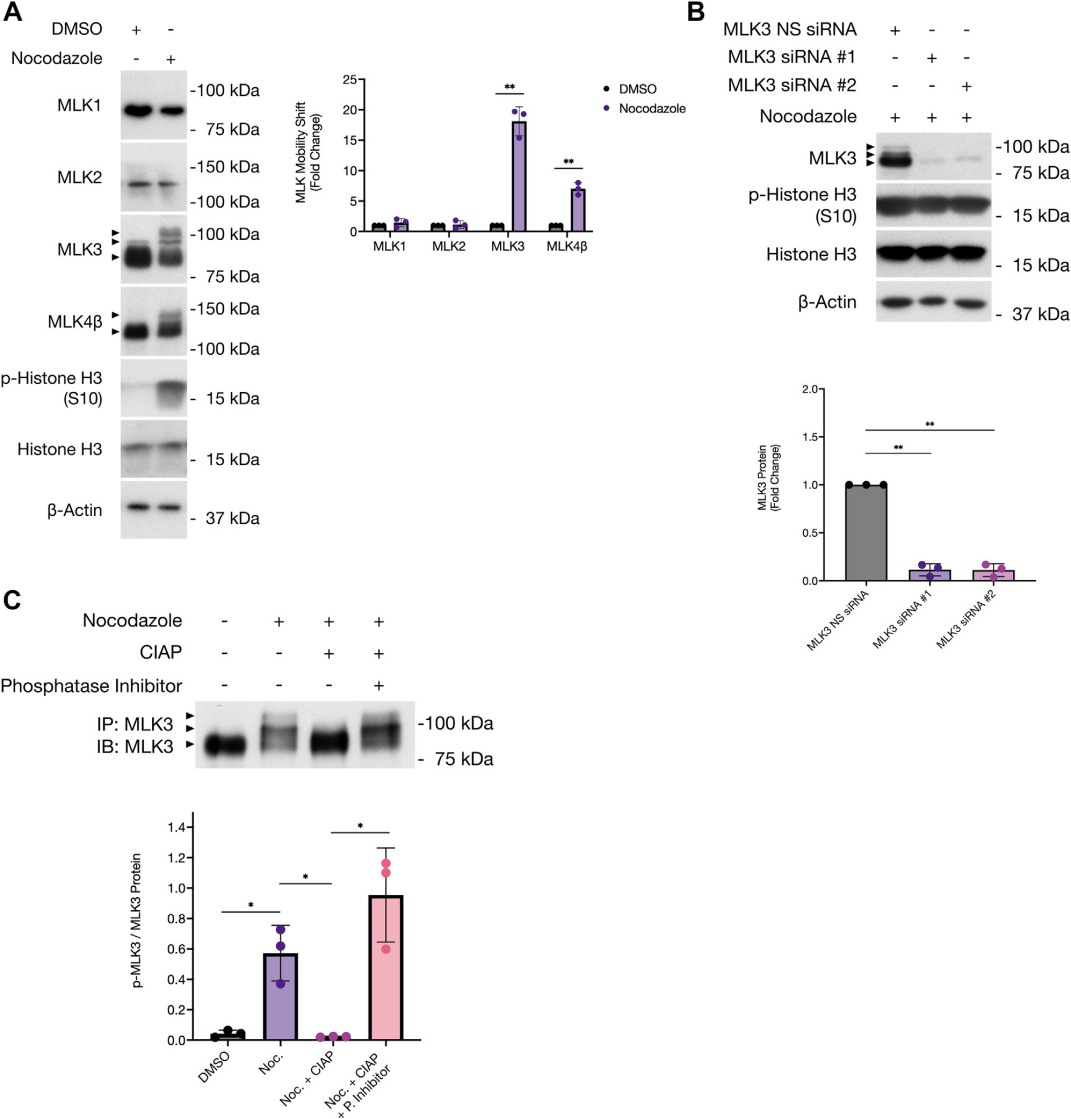
**MLK3 is phosphorylated during mitosis in SKOV3 ovarian cancer cells.***A*, SKOV3 cells were treated with nocodazole for 16 h to induce mitotic arrest. Whole-cell extracts were analyzed by SDS-PAGE and immunoblotted with the indicated antibodies. Densitometric analysis of three independent biological replicates (n = 3). *B*, knockdown of MLK3 by siRNA and nocodazole treatment was performed in SKOV3 cells, and MLK3 mobility shift was analyzed by SDS-PAGE and immunoblotted with the indicated antibodies (n = 3). *C*, SKOV3 cells were treated with nocodazole for 16 h to induce mitotic arrest. Immunoprecipitation of endogenous MLK3 was performed, and samples were treated with CIAP alone, or in combination with phosphatase inhibitor. Samples were analyzed by SDS-PAGE and immunoblotted for MLK3. Densitometric analysis of three independent biological replicates (n = 3). All results are reported as mean ± SD; ∗*p* ≤ 0.05, ∗∗*p* ≤ 0.01, ∗∗∗*p* ≤ 0.001, and ∗∗∗∗*p* ≤ 0.0001. CIAP, calf intestinal phosphatase; MLK3, mixed lineage kinase 3.

To study the regulation of MLK3 during ovarian cancer cell cycle progression, SKOV3 ovarian cancer cells were arrested in mitosis by treatment with nocodazole (1 μg/ml) for 16 h, as indicated by the increase in phospho-Histone H3 (Ser10; p-Histone H3) protein levels (Fig. 1*A*) and by flow cytometry analysis (Fig. S1). After treatment, whole cell extracts were analyzed by SDS-PAGE and immunoblotted for MLK1, MLK2, MLK3, and MLK4β proteins. Treatment with nocodazole resulted in reduced electrophoretic mobility of MLK3 and MLK4β but not MLK1 or MLK2 (Fig. 1*A*). Moreover, we confirmed the identity of the shifted bands and antibody specificity for endogenous MLK3 by knocking down MLK3 in nocodazole-treated SKOV3 cells using siRNA with two different MLK3 target sequences (Fig. 1*B*).

To test whether the MLK3 mobility shift was due to phosphorylation, phosphatase treatment of MLK3 protein was performed. SKOV3 cells were arrested in mitosis with nocodazole treatment for 16 h. Endogenous MLK3 was immunoprecipitated and treated with calf intestinal phosphatase (CIAP) alone or in combination with a phosphatase inhibitor. In nocodazole-treated cells, CIAP alone prevented the MLK3 mobility shift (Fig. 1*C*). In contrast, treatment with CIAP and phosphatase inhibitor did not block the MLK3 mobility shift, suggesting that MLK3 mobility shift is due to phosphorylation in nocodazole-treated cells. Taken together, our results demonstrate that MLK3 is phosphorylated during mitosis in ovarian cancer cells.

### CDK1 inhibition blocked MLK3 phosphorylation in nocodazole-treated cells

**Figure 2:**
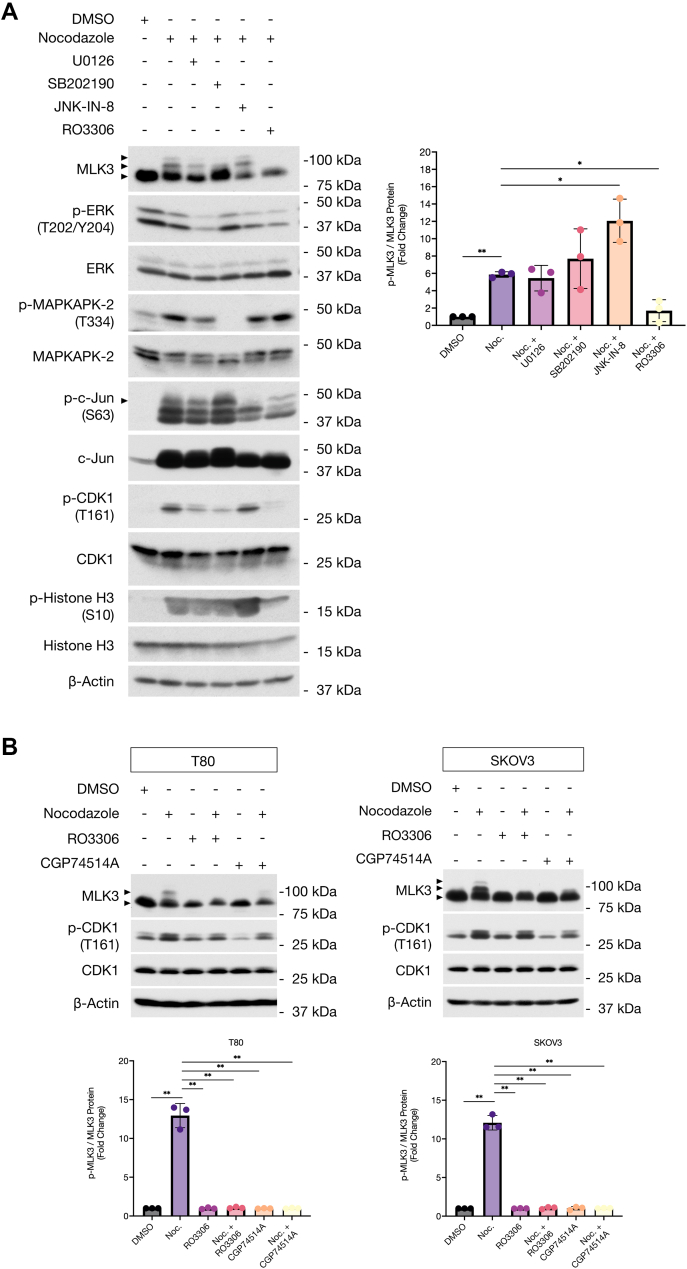
**MLK3 phosphorylation is blocked by CDK1 inhibition in T80 normal ovarian cells and SKOV3 ovarian cancer cells.***A*, SKOV3 cells were treated with nocodazole for 16 h to induce mitotic arrest and treated with different kinase inhibitors as indicated. Samples were analyzed by SDS-PAGE and immunoblotted for indicated antibodies (n = 3). *B*, T80 and SKOV3 cells were treated with RO3306 or CGP74514A alone or in combination with nocodazole (30 min RO3306 or CGP74514A treatment). Samples were analyzed by immunoblotting for the indicated proteins. All densitometric analyses results represent three independent biological replicates (n = 3). Results are reported as mean ± SD. ∗*p* ≤ 0.05, ∗∗*p* ≤ 0.01, ∗∗∗*p* ≤ 0.001, and ∗∗∗∗*p* ≤ 0.0001. CDK1, cyclin-dependent kinase 1; MLK3, mixed lineage kinase 3.

Based on our results from Figure 1, we concluded that MLK3 is phosphorylated in mitotic SKOV3 cells. To investigate the kinase responsible for MLK3 phosphorylation during mitosis, SKOV3 cells were treated with nocodazole in combination with different MAPK inhibitors. A CDK1 inhibitor was also included since CDK1 is a critical regulator of G2/M transition (33, 50). SKOV3 cells were treated with the MEK inhibitor U0126 (100 μM), p38 inhibitor SB202190 (10 μM), JNK inhibitor JNK-IN-8 (0.1 μM), or with the CDK1 inhibitor RO3306 (9 μM), in combination with nocodazole (1 μg/ml) for 16 h. We observed that phosphorylation of MLK3 in mitotic cells was blocked by the CDK1 inhibitor, but not with the ERK, p38, or JNK inhibitors (Fig. 2*A*), which suggests that CDK1 is the kinase responsible for MLK3 phosphorylation during mitosis.

To confirm that treatment with nocodazole and RO3306 for a prolonged period did not result in cells exiting mitosis and prematurely entering G1 phase, T80 normal ovarian cells and SKOV3 ovarian cells were treated with nocodazole for 16 h, and then RO3306 or CGP74514A (CDK1 inhibitor) was added to the cells in the final 30 min of the 16 h nocodazole treatment. As expected, T80 and SKOV3 cells treated with RO3306 or CGP74514A alone for 30 min showed reduced phospho-CDK1 (Thr161; p-CDK1). In contrast, T80 and SKOV3 cells treated with nocodazole and RO3306 or CGP74514A had increased levels of p-CDK1, indicating that the cells did not exit M phase and entered G1 phase (Fig. 2*B*). We observed that CDK1 inhibition with RO3306 or CGP74514A blocks MLK3 phosphorylation in M phase cells in both normal and ovarian cancer cells (Fig. 2*B*). Similar results were observed in T29 normal ovarian cells and TOV112D and HEY ovarian cancer cells (Fig. S2). Therefore, we propose that MLK3 phosphorylation in early M phase is mediated by CDK1.

### CDK1 and CDK2 phosphorylate MLK3

**Figure 3:**
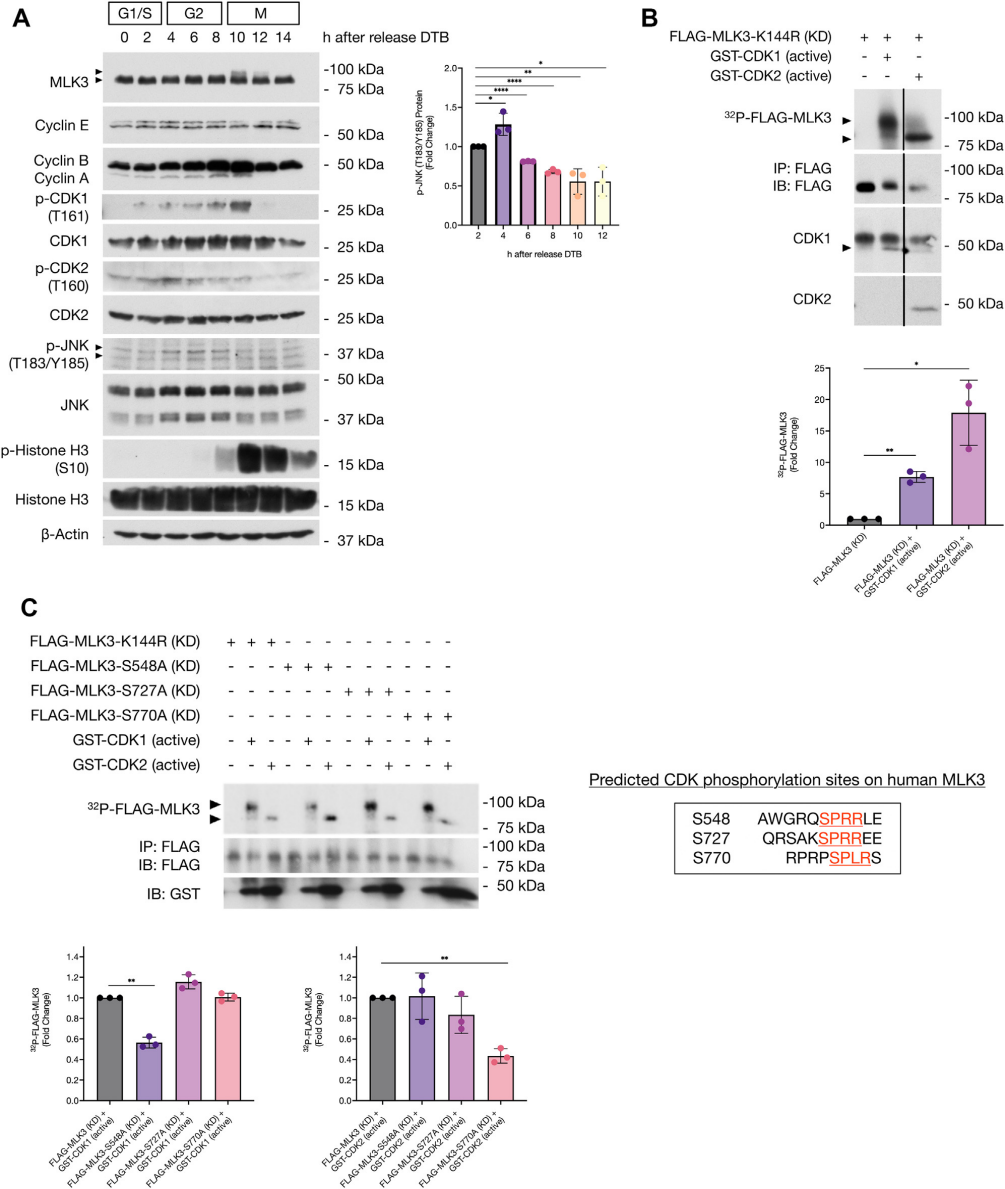
**MLK3 is phosphorylated by CDK1 and CDK2.***A*, SKOV3 cells were synchronized by double thymidine block (DTB). Whole-cell extracts of different time points after DTB release were analyzed by SDS-PAGE and immunoblotted with the indicated antibodies against several cell cycle markers and protein kinases (n = 3). *B*, [γ^32^P] ATP *in vitro* kinase assays were performed with FLAG-MLK3-K144R kinase-dead (KD) immunoprecipitates from HEK293 cells, in the presence or the absence of purified active GST-CDK1/cyclin A and active GST-CDK2/cyclin A. Samples were analyzed by SDS-PAGE, developed by autoradiography, and followed by immunoblotting of indicated antibodies (n = 3). Lanes 2 and 3 are noncontiguous lanes from the same immunoblot. *C*, in *red* and *underlined* are shown the CDK phosphorylation consensus sequences and residues that are found on human MLK3 corresponding to the CDK consensus sequence S/T-P-X-K/R. [γ^32^P] ATP *in vitro* kinase assays were performed with FLAG-MLK3-K144R (KD), FLAG-MLK3-S548A (KD), FLAG-MLK3-S727A (KD), and FLAG-MLK3-S770A (KD) immunoprecipitates from HEK293 cells in the presence or the absence of purified active GST-CDK1/cyclin A and active GST-CDK2/cyclin A. Samples were analyzed by SDS-PAGE, developed by autoradiography, and followed by immunoblotting of indicated antibodies (n = 3). All densitometric analyses results represent three independent biological replicates (n = 3). Results are reported as mean ± SD; ∗*p* ≤ 0.05, ∗∗*p* ≤ 0.01, ∗∗∗*p* ≤ 0.001, and ∗∗∗∗*p* ≤ 0.0001. CDK1, cyclin-dependent kinase 1; CDK2, cyclin-dependent kinase 2; GST, glutathione-*S*-transferase; HEK293, human embryonic kidney 293 cell line; MLK3, mixed lineage kinase 3.

To analyze the regulation of MLK3 during the cell cycle in ovarian cancer cells without the addition of kinase inhibitors or nocodazole, SKOV3 cells were synchronized at the G1/S boundary by performing a double thymidine block (DTB). SKOV3 cells were treated with thymidine (2 mM) and incubated for 18 h. Cells were then released for 9 h and blocked again with 2 mM thymidine for 18 h. After the second thymidine treatment, cells were released and collected at 0, 2, 4, 6, 8, 10, 12, and 14 h. Phosphorylation of MLK3 was first observed in late G2/early M phase in SKOV3 cells (Fig. 3*A*) and TOV112D and HEY cells (Fig. S3), concurrent with the increase in CDK1/cyclin A and CDK1/cyclin B activity. We also observed increased levels of phospho-JNK (Thr183/Tyr185; p-JNK) in late G1/S and G2 phases, and a decrease in late G2/early M phase, which is concurrent with MLK3 phosphorylation in early M phase (Fig. 3*A*).

To investigate whether CDK1 and CDK2 can phosphorylate MLK3 *in vitro*, we performed an *in vitro* [γ^32^P] ATP kinase assay with active glutathione-*S*-transferase (GST)-CDK1/cyclin A and GST-CDK2/cyclin A. FLAG-MLK3-K144R kinase dead (KD) expressed in human embryonic kidney 293 (HEK293) cells used as substrate for the kinase assay. We observed an increase in MLK3 phosphorylation in the presence of CDK1 and CDK2 (Fig. 3*B*), suggesting that these protein kinases can phosphorylate MLK3 *in vitro*. In addition, we also observed that phosphorylation of MLK3 by CDK1, and not CDK2, promoted MLK3 mobility shift, which is consistent with the observations in synchronized cells with DTB and in nocodazole-treated cells.

Because our results demonstrated that CDK1 and CDK2 can phosphorylate MLK3 *in vitro*, we investigated the specific CDK phosphorylation site(s) on human MLK3. Previous MALDI-reTOF MS analysis of MLK3 phosphorylation sites showed that MLK3 might be a target for proline-directed kinases (51). We identified three potential phosphorylation sites that are part of the CDK phosphorylation consensus sequence Ser/Thr-Pro-X-Lys/Arg (52, 53, 54). The three potential phosphorylation sites are Ser548 (Ser-Pro-Arg-Arg), Ser727 (Ser-Pro-Arg-Arg), and Ser770 (Ser-Pro-Leu-Arg) (Fig. 3*C*). To identify whether these residues are specifically phosphorylated by CDK1 or CDK2, we first created single phosphomutants using site-directed mutagenesis by substituting the amino acid Ser for Ala in an MLK3 KD DNA template (FLAG-MLK3-K144R). The FLAG-MLK3 proteins were expressed in HEK293 cells, immunoprecipitated, and used as substrates in an *in vitro* [γ^32^P] ATP kinase assay with active GST-CDK1/cyclin A and GST-CDK2/cyclin A. In the presence of active CDK1/cyclin A, phosphorylation of FLAG-MLK3 was only reduced when Ser548 was mutated to Ala. In the presence of active CDK2/cyclin A, phosphorylation of FLAG-MLK3 was only reduced when Ser770 was mutated to Ala (Fig. 3*C*). We observed that mutation of Ser727 to Ala did not affect MLK3 phosphorylation by CDK1 or CDK2 (Fig. 3*C*). We also observed that phosphorylation of FLAG-MLK3-S548A and FLAG-MLK3-S770A (shifted bands) was reduced when compared with FLAG-MLK3 WT (Fig. S4), suggesting that both phosphorylation sites contribute to MLK3 mobility shift on SDS-PAGE. Taken together, our results indicate that CDK1 phosphorylates MLK3 *in vitro* on Ser548 and that CDK2 phosphorylates MLK3 *in vitro* on Ser770.

### Phosphorylation of MLK3 by CDK1 and CDK2 affects MLK3 and JNK activity during G1/S, G2, and M phases

**Figure 4:**
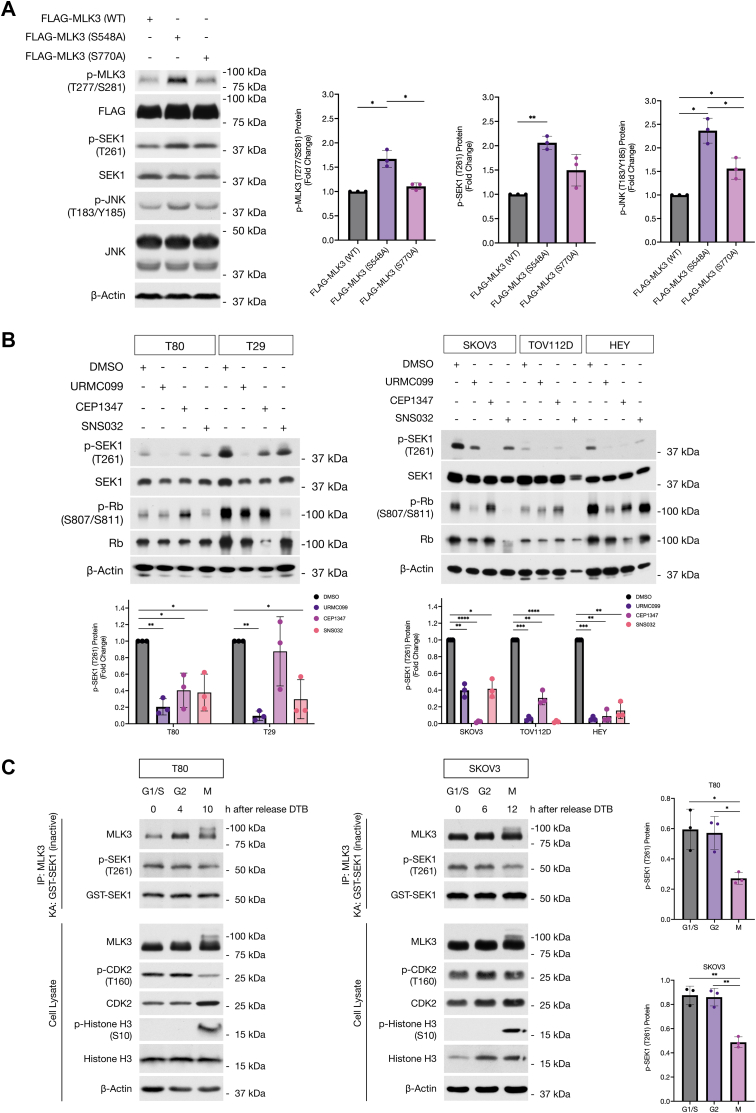
**MLK3 phosphorylation by CDK1 and CDK2 during G1/S, G2, and M phases affects MLK3 and JNK activities.***A*, FLAG-MLK3-WT, FLAG-MLK3-S548A, and FLAG-MLK3-S770A were expressed in HEK293 cells. After 48 h of transfection, HEK293 cells were lysed and whole-cell extracts were analyzed by immunoblotting to assessed MLK3 activity. MLK3 activity was measured by phospho-MLK3 (Thr277/Ser281), phospho-SEK1 (Thr261), and phospho-JNK (Thr183/Tyr185) protein levels (n = 3). *B*, T80, T29, SKOV3, TOV112D, and HEY cells treated with DMSO, URMC099, CEP1347, or SNS032 for 24 h. Whole-cell extracts were analyzed by SDS-PAGE and immunoblotted with the indicated antibodies (n = 3). *C*, immunoblot of GST-SEK1 (inactive) kinase assay using endogenous MLK3 immunoprecipitates from G1/S, G2, and M phases in synchronized T80 and SKOV3 cells. KA stands for kinase assay (n = 3). All densitometric analyses represent three independent biological replicates (n = 3). Results are reported as mean ± SD. ∗*p* ≤ 0.05, ∗∗*p* ≤ 0.01, ∗∗∗*p* ≤ 0.001, and ∗∗∗∗*p* ≤ 0.0001. CDK1, cyclin-dependent kinase 1; CDK2, cyclin-dependent kinase 2; DMSO, dimethyl sulfoxide; GST, glutathione-*S*-transferase; HEK293, human embryonic kidney 293 cell line; JNK, c-Jun N-terminal kinase; MLK3, mixed lineage kinase 3.

Based on the results of the DTB of SKOV3 cells and the differences in JNK activity in late G1/S, early G2, and M phases, we hypothesized that CDK2 (during early G2 phase) and CDK1 (during early M phase) plays an essential role in regulating MLK3 and JNK activity in ovarian cancer cells. To further test this possibility, HEK293 cells were transfected with FLAG-MLK3 WT, FLAG-MLK3-S548A, or FLAG-MLK3-S770A, and the amounts of phosphorylated, activated MLK3, SEK1, and JNK were assessed with phospho-MLK3 (Thr277/Ser281; p-MLK3), phospho-SEK1 (Thr261; p-SEK1), and p-JNK (Thr183/Tyr185) antibodies. In cells that expressed FLAG-MLK3-S548A, we observed a 1.6-fold increase in activated p-MLK3, 2.1-fold increase in p-SEK1, and 2.4-fold increase in p-JNK when compared with cells expressing FLAG-MLK3 (WT) (Fig. 4*A*). Cells that expressed FLAG-MLK3-S770A showed no significant difference in p-MLK3 and p-SEK1, when compared with WT cells (Fig. 4*A*). These results suggest that phosphorylation of MLK3 on Ser548 negatively regulates its ability to activate JNK.

To further explore the hypothesis that CDK1 and CDK2 regulate MLK3 and JNK activation in ovarian epithelial cells, we treated T80, T29, SKOV3, TOV112D, and HEY cells with dimethyl sulfoxide (DMSO), MLK3 inhibitors URMC099 and CEP1347, and CDK2 inhibitor SNS032 for 24 h (Fig. 4*B*). The URMC099 and CEP1347 inhibitors used in this study have been previously identified as selective inhibitors for MLK3 (55, 56). We observed that p-SEK1 was substantially reduced by SNS032 (Fig. 4*B*), suggesting that CDK2 is an important regulator of MLK3 and SEK1–JNK activities. In addition, we treated T80 and SKOV3 cells with DMSO, URMC099, SNS032, and RO3306 for 24 h and did not observe a substantial decrease in p-SEK1 levels when CDK1 was inhibited by RO3306 (Fig. S5). To validate these observations, T80 and SKOV3 cells were synchronized by DTB and collected at the indicated time points after DTB release. Endogenous MLK3 was then immunoprecipitated from the cells that were collected after release from DTB, representing a population of cells at G1/S, G2, and M phases as indicated by immunoblotting of cell cycle markers against active p-CDK2 and p-Histone H3 (Fig. 4*C*) and by flow cytometry analysis (Fig. S6). The MLK3 immunoprecipitates from cells in different stages of the cell cycle were used in an *in vitro* kinase assay with GST-SEK1 (inactive) as substrate. MLK3 immunoprecipitated from M phase cells exhibited a mobility shift and had reduced kinase activity toward SEK1 (as demonstrated by the reduced amount of p-SEK1) in comparison to MLK3 immunoprecipitated from cells in G1/S and G2 phases (Fig. 4*C*). Overall, these results suggest that phosphorylation of MLK3 by CDK2 enhances the activity of MLK3 during G1/S and G2 phases, whereas phosphorylation of MLK3 by CDK1 decreases MLK3 activity during M phase in normal and cancerous ovarian epithelial cells.

### MLK3 inhibition blocked cell proliferation and caused a cell cycle arrest in ovarian cancer cells

**Figure 5:**
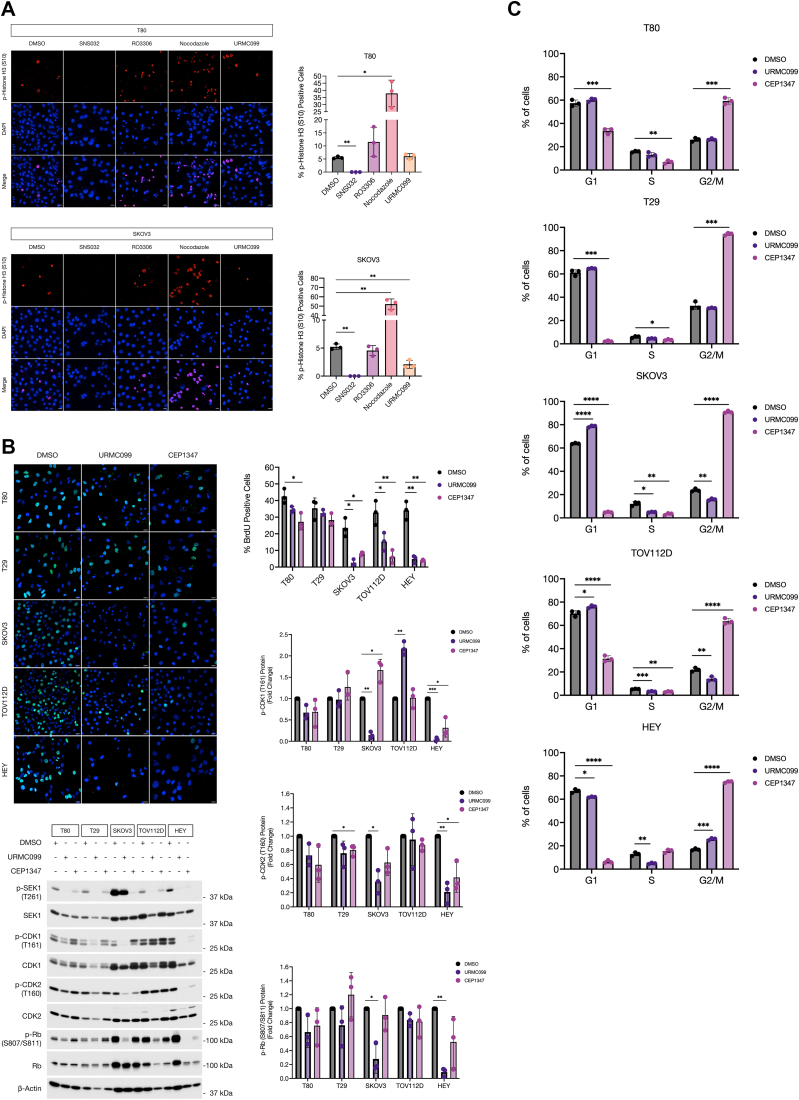
**MLK3 inhibition causes cell cycle arrest in ovarian cancer cells.***A*, percentage of phospho-Histone H3 (Ser10)–positive cells in T80 and SKOV3 cells treated with the indicated inhibitors for 24 h. Immunostaining for phospho-Histone H3 (Ser10) is shown in *red* and DNA staining in *blue*. The scale bar represents 20 μm. Data are the results of three independent biological replicates (n = 3) where at least 200 cells were counted per condition. *B*, percentage of BrdU-positive cells in T80, T29, SKOV3, TOV112D, and HEY cells treated with DMSO, URMC099, or CEP1347 for 24 h. BrdU staining is shown in *green* and DNA in *blue*. The scale bar represents 20 μm. Data are the results of three independent biological replicates (n = 3) where at least 200 cells were counted per condition. Whole-cell extracts from the BrdU assay were analyzed by immunoblot with the indicated antibodies. All densitometric analyses represent three independent biological replicates (n = 3). *C*, flow cytometry analysis of T80, T29, SKOV3, TOV112D, and HEY cells treated with DMSO, URMC099, or CEP1347 for 24 h. All results represent three independent biological replicates (n = 3). Results are reported as mean ± SD. ∗*p* ≤ 0.05, ∗∗*p* ≤ 0.01, ∗∗∗*p* ≤ 0.001, and ∗∗∗∗*p* ≤ 0.0001. BrdU, bromodeoxyuridine; DMSO, dimethyl sulfoxide; MLK3, mixed lineage kinase 3.

We have demonstrated that MLK3 and JNK activities are differentially regulated by CDK1 and CDK2. To determine the effect of inhibiting MLK3 activity on cell proliferation, we performed p-Histone H3 immunofluorescence staining to detect mitotic cells in T80 and SKOV3 cells treated with DMSO, SNS032, RO3306, nocodazole, and URMC099 for 24 h. We observed a significant reduction in the percentage of p-Histone H3–positive cells in SKOV3 cells treated with URMC099 but not in T80 cells (Fig. 5*A*), suggesting that MLK3 might play an essential role in ovarian cancer cell cycle progression. To confirm these observations, a bromodeoxyuridine (BrdU) assay was performed with T80, T29, SKOV3, TOV112D, and HEY cells treated with DMSO, URMC099, or CEP1347. After 24 h treatment, immunofluorescence staining with BrdU antibody showed no difference in cell proliferation when T80 and T29 normal ovarian cells were treated with URMC099; however, T80 cells treated with CEP1347 showed decreased cell proliferation (Fig. 5*B*). In contrast, SKOV3, TOV112D, and HEY ovarian cancer cells treated with URMC099 or CEP1347 exhibited a significant reduction in cell proliferation as shown by the reduced percentage of BrdU-positive cells (Fig. 5*B*). These results indicate that MLK3 activity is required for cell proliferation of ovarian cancer cells but not in normal ovarian epithelial cells.

To further characterize the reduction in cell proliferation caused by MLK3 inhibition, whole cell extracts from the BrdU assay were immunoblotted for p-CDK1 (Thr161), phospho-CDK2 (Thr160; p-CDK2), and phospho-Rb (retinoblastoma protein; Ser807/Ser811; p-Rb). Interestingly, we observed that SKOV3 and HEY but not TOV112D treated with URMC099 had a significant decrease in active p-CDK1 in comparison to cells treated with DMSO. Moreover, SKOV3 and HEY, but not TOV112D treated with URMC099, exhibited reduced p-CDK2 and p-Rb protein levels when compared with the DMSO control (Fig. 5*B*). Unlike SKOV3, TOV112D, and HEY ovarian cancer cells, T80 and T29 normal ovarian epithelial cells did not exhibit decreased levels of p-CDK1, p-CDK2, or p-Rb when treated with URMC099 (Fig. 5*B*). These results suggest that MLK3 inhibition by URMC099 causes a G1/S arrest in SKOV3, TOV112D, and HEY ovarian cancer cells. In contrast, we observed that CEP1347 treatment in SKOV3, but not in TOV112D and HEY cells, resulted in a significant increase in active p-CDK1 in comparison to cells treated with DMSO. Moreover, CEP1347 treatment in SKOV3 and HEY, but not in TOV112D cells, led to a reduction in p-CDK2; whereas HEY, but not SKOV3 and TOV112D cells, had reduced p-Rb protein levels when compared with the DMSO control (Fig. 5*B*). T80 and T29 cells did not have a substantial decrease in the levels of p-CDK1, p-CDK2, or p-Rb when treated with CEP1347 (Fig. 5*B*). These results confirm that MLK3 inhibition by CEP1347 causes a G2/M arrest in T80, T29, SKOV3, TOV112D, and HEY cells.

Flow cytometric analysis of SKOV3, TOV112D, and HEY cells treated with URMC099 showed a significant increase in the population of cells in G1 phase and a significant decrease in the S and G2/M phases when compared with DMSO-treated cells, which indicates a G1/S cell cycle arrest (Fig. 5*C*). Moreover, G1/S arrest was not observed in T80 and T29 cells treated with URMC099 (Fig. 5*C*). T80, T29, SKOV3, TOV112D, and HEY cells treated with CEP1347 showed a significant increase in the population of cells in G2/M phase and a significant decrease in the G1 and S phases when compared with DMSO-treated cells, which indicates a G2/M cell cycle arrest (Fig. 5*C*). Overall, inhibition of MLK3 resulted in cell cycle arrest and decreased cell proliferation in SKOV3, TOV112D, and HEY ovarian cancer cells, which underscores the importance of MLK3 activity in ovarian cancer cell cycle progression.

## Discussion

**Figure 6:**
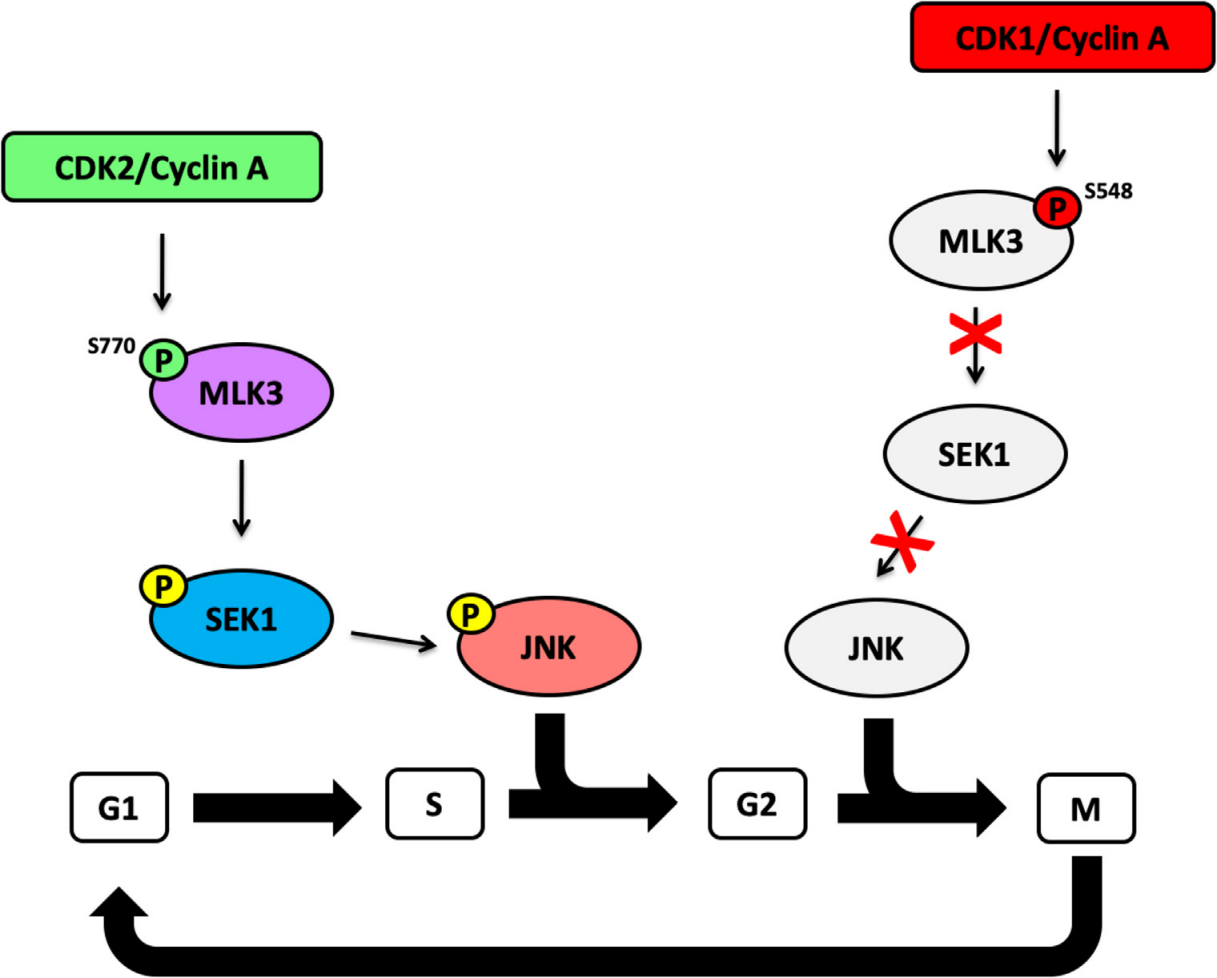
**Proposed model for the regulation of MLK3 by CDK1 and CDK2 in ovarian cancer cell cycle progression.** Phosphorylation of MLK3 by CDK2 on Ser770 during late G1/S phase increases MLK3 activity, which causes activation of the JNK pathway. In late G2 and early M phases, phosphorylation of MLK3 by CDK1 on Ser548 inhibits MLK3 activity and causes inactivation of the JNK pathway, thereby allowing ovarian cancer cells to progress through the cell cycle. CDK1, cyclin-dependent kinase 1; CDK2, cyclin-dependent kinase 2; JNK, c-Jun N-terminal kinase; MLK3, mixed lineage kinase 3.

Understanding how CDKs and MAPKs are regulated in different cellular processes is critical as deregulation of these protein kinases is characteristic of many diseases including cancer. Previous studies in our laboratory have demonstrated that MLK3 is required for invasion and proliferation of ovarian cancer cells (4, 6, 57); however, the role of MLK3 in ovarian cancer cell cycle progression has not been elucidated. Our results demonstrate that during late G1/S and early G2 phases, activated CDK2/cyclin A phosphorylates MLK3 on Ser770 causing an increase in MLK3 activity and activation of its downstream targets SEK1 and JNK. In late G2 and early M phases, activated CDK1/cyclin A phosphorylates MLK3 on Ser548, promoting an inhibitory phosphorylation that causes a decrease in MLK3 and JNK activities to allow proper G2/M transition. We propose a model in which CDK1 and CDK2 phosphorylate MLK3, and this phosphorylation works as a “on/off” switch that in turn regulates MLK3 and JNK activity to control cell cycle progression in ovarian cancer cells (Fig. 6).

The regulation of a MAP3K by a CDK has been demonstrated previously for the MAP3K, B-Raf. Borysov *et al.* (58) showed in *Xenopus* egg extracts that CDK1/cyclin B can directly phosphorylate and activate B-Raf. Furthermore, phosphorylation of *Xenopus* B-Raf on Ser144 by CDK1/cyclin B resulted in activation of B-Raf–MEK–ERK signaling during M phase (58). These findings demonstrate that a CDK can phosphorylate and regulate the activity of a MAP3K and its downstream MAPK signaling pathway. This is consistent with our findings that CDK1 and CDK2 phosphorylate and regulate MLK3 activity and MLK3-dependent activation of JNK signaling. Therefore, our work uncovers a novel mechanism in mammalian cells in which the activity of a MAP3K is regulated by a CDK.

In previous studies with HeLa cells, MLK inhibition resulted in a G2/M arrest, and MLK3 was found to be important for microtubule stability during G2/M transition (48, 59). In the study by Cha *et al.* (48), MLK inhibition was achieved with the MLK inhibitor, CEP-11004. Consistent with our results, treatment of HeLa cells with CEP-11004 caused a G2/M arrest because cells were unable to exit mitosis. In addition, another study by Wang *et al.* (49) also showed that inhibition of MLK3 with CEP1347 resulted in a G2/M arrest of estrogen receptor–positive breast cancer cells. In ovarian cancer cells treated with the MLK3 inhibitor, URMC099, we showed that inhibition of MLK3 activity caused a decrease in cell proliferation and a G1/S arrest. In contrast, inhibition of MLK3 by CEP1347 caused a G2/M arrest in both normal and ovarian cancer cells. It is important to note that CEP-11004 and CEP1347 can inhibit other members of the MLK family, which perhaps can explain why treatment with these inhibitors affected normal cells and also caused a different type of cell cycle arrest (60). We previously demonstrated that MLK3 activity and expression are higher in ovarian cancer cells when compared with normal ovarian epithelial cells (4). Therefore, we propose that ovarian cancer cells are more dependent on MLK3 activity than normal ovarian cells to be able to proliferate and progress through the cell cycle.

Several studies in fibroblasts and ovarian granulosa cells demonstrated that JNK is important for entry into mitosis by regulating Aurora B and Histone H3 phosphorylation at Ser10 (43, 44); however, it remains unclear how JNK activity itself is regulated during the cell cycle. Many of the previous studies regarding JNK activity in cell division were focused on the JNK–c-Jun axis and its downstream targets. Our results elucidate the mechanism by which JNK activity is regulated during cell division by its major upstream activator, MLK3. We showed that CDK1 and CDK2 regulate MLK3 to control JNK activity during cell cycle progression in human ovarian cancer cells. These results reveal that tight control of MLK3 and JNK activities by CDK1 and CDK2 during the cell cycle is essential for proliferation of ovarian cancer cells. Overall, our previous and current findings demonstrate essential functions for MLK3 in ovarian cancer cell proliferation, invasion, and cell cycle progression and suggest that inhibition of MLK3 activity could be a potential therapeutic approach for the treatment of ovarian cancer.

## Experimental procedures

### Cell culture, treatments, and synchronization

T80 and T29 immortalized normal ovarian epithelial cells and HEY ovarian cancer cells were provided by Dr Jinsong Liu (UT MD Anderson Cancer Center). T80 and T29 were cultured at 37 °C with 5% CO_2_ in M-199 media supplemented with 15% calf serum, MCDB-105, l-glutamine, and penicillin/streptomycin (61). Human ovarian cancer (SKOV3, TOV112D, and HEY) and HEK293 cell lines were acquired from the American Type Culture Collection. SKOV3, TOV112D, HEY, and HEK293 cells were cultured at 37 °C with 5% CO_2_ in Dulbecco's modified Eagle's medium supplemented with 10% calf serum, l-glutamine, and penicillin/streptomycin. Cell treatments were performed as indicated with DMSO as vehicle or with the following inhibitors: 1 μg/ml nocodazole (catalog no.: 487928; MilliporeSigma), 100 μM U0126 (catalog no.: 70970; Cayman Chemical), 10 μM SB202190 (catalog no.: S7067; MilliporeSigma), 0.1 μM JNK-IN-8 (catalog no.: SML1246; MilliporeSigma), 9 μM RO3306 (catalog no.: 15149; Cayman Chemical), 10 μM CGP74514A (catalog no.: 300344; Santa Cruz Biotechnology), 3 μM (for SKOV3) or 10 μM (for T80, T29, TOV112D, and HEY), URMC099 (catalog no.: HY-12599; MedChemExpress), 10 μM CEP1347 (catalog no.: 4924; Tocris Bioscience), and 10 μM (for SKOV3, TOV112D, and HEY) or 1 μM (for T80 and T29) SNS032 (catalog no.: 17904; Cayman Chemical). T80, SKOV3, TOV112D, and HEY cells were synchronized using 2 mM thymidine (catalog no.: 226740050; Thermo Fisher Scientific) as previously described (62) and collected after release from DTB at the indicated time points.

### Phosphatase treatment

SKOV3 cells were either untreated or arrested in mitosis with 1 μg/ml nocodazole for 16 h, and endogenous MLK3 was immunoprecipitated using MLK3 (D-11) antibody (catalog no.: 166639; Santa Cruz Biotechnology) and Protein A/G PLUS-Agarose beads (catalog no.: 2003; Santa Cruz Biotechnology). MLK3–nocodazole immunoprecipitates were either untreated or treated with calf intestinal alkaline phosphatase (CIAP) (catalog no.: 18009027; Invitrogen) alone, or in combination with CIAP and phosphatase inhibitor cocktail 3 (catalog no.: P0044; MilliporeSigma) for 1 h at 37 °C. The reaction was stopped with 1× SDS sample buffer and boiled for 5 min at 95 °C. Samples were then analyzed by immunoblotting as indicated.

### Immunoblotting

Whole cell extracts were prepared and separated by 10% or 12.5% SDS-PAGE. Proteins were transferred to a polyvinylidene difluoride membrane (catalog no.: IPVH00010; MilliporeSigma) and blocked with 5% nonfat dry milk. Immunoblotting was performed using the following primary antibodies: MLK1 (catalog no.: 50295), p-Histone H3 (Ser10) (catalog no.: 9701), phospho-ERK (Thr202/Tyr204) (catalog no.: 4370), p-JNK (Thr183/Tyr185) (catalog no.: 9251), phospho-p38 (Thr180/Tyr182) (catalog no.: 9211), phospho-MAPKAPK-2 (Thr334) (catalog no.: 3007), MAPKAPK-2 (catalog no.: 3042), phospho-c-Jun (Ser63) (catalog no.: 9261), p-CDK1 (Thr161) (catalog no.: 9114), p-CDK2 (Thr160) (catalog no.: 2561), p-SEK1 (Thr261) (catalog no.: 9151), SEK1 (catalog no.: 9152), and GST tag (catalog no.: 2625) from Cell Signaling Technology; MLK3 (H-3) (catalog no.: sc-166592), β-actin (C4) (catalog no.: sc-47778), Histone H3 (catalog no.: sc-517576), ERK (C-9) (catalog no.: sc-514302), JNK (D-2) (catalog no.: sc-7345), p38 (A-12), (catalog no.: sc-7972), c-Jun (G-4) (catalog no.: sc-74543), p-CDK1 (Tyr15) (catalog no.: sc-136014), CDK1 (catalog no.: sc-54), CDK2 (D-12) (catalog no.: sc-6248), p-Rb (Ser807/Ser811) (catalog no.: sc-16670), Rb (catalog no.: sc-102), cyclin E (M-20) (catalog no.: sc-481), cyclin B (catalog no.: sc-245), cyclin A (catalog no.: sc-239), and GST tag (catalog no.: sc-138) from Santa Cruz Biotechnology; MLK2 (catalog no.: 19974-1-AP) from Proteintech; MLK4β (catalog no.: NBP1-41081) from Novus Biologicals; p-MLK3 (Thr277/Ser281) (catalog no.: PA5-105817) from Invitrogen; and FLAG tag (catalog no.: 200474) from Agilent Technologies. Membranes were incubated with the appropriate horseradish peroxidase–conjugated secondary antibodies: Immun-Star Goat Antimouse (catalog no.: 1705047; Bio-Rad), Immun-Star Goat Anti-Rabbit (catalog no.: 1705046; Bio-Rad), and Goat Antirat (catalog no.: 31470; Invitrogen). The membranes were then developed with Immobilon chemiluminescent detection solutions (catalog no.: WBKLS0500; MilliporeSigma) and exposed to an X-ray film.

### Site-directed mutagenesis

Site-directed mutagenesis of human MLK3 was performed using the QuikChange Lightning Site-directed Mutagenesis kit (catalog no.: 210519; Agilent Technologies). Phosphomutants were generated in FLAG-MLK3 WT and FLAG-MLK3-K144R KD complementary DNA templates using the following oligonucleotide sequences: S548A mutant (sense 5’-GCATGGGGCCGCCAGGCCCCCCGACGTCTGGAG-3′, antisense 5’-CTCCAGACGTCGGGGGGCCTGGCGGCCCCATGC-3′); S727A mutant (5’-CAGCGGTCAGCCAAGGCCCCCCGACGTGAGGAG-3′, antisense 5’-CTCCTCACGTCGGGGGGCCTTGGCTGACCGCTG-3′); S770A mutant (sense 5’-CGACCTCGGCCCGCGCCCCTTCGCAGC-3′, antisense 5’-GCTGCGAAGGGGCGCGGGCCGAGGTCG-3′); underlined base pairs represent mismatch with WT MLK3 template. Phosphomutants were verified by Sanger sequencing (Genewiz).

### Plasmid transfections

For [γ^32^P] ATP *in vitro* kinase assays, FLAG-MLK3-K144R (KD), FLAG-MLK3-S548A (KD), FLAG-MLK3-S727A (KD), and FLAG-MLK3-S770A (KD) were transfected into HEK293 cells using PolyJet (catalog no.: SL100688; SignaGen Laboratories) for 24 h. To assess MLK3 activity though SEK1 phosphorylation, FLAG-MLK3-WT, FLAG-MLK3-S548A, and FLAG-MLK3-S770A were transfected into HEK293 cells using Lipofectamine 2000 (catalog no.: 11668019; Invitrogen) for 48 h. For knockdown of MLK3 by siRNA, nonspecific siRNA and two different MLK3 siRNA target sequences were transfected into SKOV3 cells using Lipofectamine 2000 as previously described (63).

### *In vitro* kinase assay

FLAG-MLK3-K144R (KD), FLAG-MLK3-S548A (KD), FLAG-MLK3-S727A (KD), and FLAG-MLK3-S770A (KD) constructs were transfected into HEK293 cells and immunoprecipitated using FLAG-agarose beads (catalog no.: A2220; MilliporeSigma). Immunoprecipitates were then suspended in kinase assay buffer (20 mM Mops [pH 7.2], 2 mM EGTA [pH 8.1], 10 mM MgCl_2_, 1 mM DTT, and 0.1% Triton X-100) with 100 μM unlabeled ATP, 10 mM MgCl_2_, and 5 μCi of [γ^32^P] ATP (PerkinElmer Health Sciences) (15). Active full-length recombinant GST-CDK1/cyclin A (0.01 μg) (catalog no.: C22-18G; SignalChem) or GST-CDK2/cyclin A (0.01 μg) (catalog no.: C29-10G; SignalChem) proteins were added to the appropriate sample and incubated for 30 min at 30 °C. The reaction was stopped with 1× SDS sample buffer and boiled for 5 min at 95 °C. Samples were then analyzed by autoradiography and immunoblotted with the indicated antibodies. For SEK1 kinase assay in T80 and SKOV3 cells, endogenous MLK3 was immunoprecipitated from cell extracts in the indicated phases of the cell cycle in synchronized cells. Endogenous MLK3 immunoprecipitates were suspended in kinase assay buffer with 100 μM unlabeled ATP, 10 mM MgCl_2_, 0.05 μg inactive GST-SEK1 recombinant protein (catalog no.: 14-378; Millipore) and incubated for 30 min at 30 °C. The reaction was stopped with 1× SDS sample buffer, boiled for 5 min at 95 °C, and analyzed by immunoblotting.

### Immunofluorescence

For p-Histone H3 (Ser10) immunostaining, T80 and SKOV3 cells were plated on glass coverslips with poly-l-lysine coating (catalog no.: GG-12-1.5-PLL; Neuvitro). Cells were treated with the indicated inhibitors for 24 h, fixed for 15 min with 4% paraformaldehyde in 1× PBS, and permeabilized for 7 min with 0.1% Triton X-100 in 1× PBS. Fixed samples were washed three times with 1× PBS and blocked overnight with 5% bovine serum albumin (BSA) in 1× PBS at 4 °C. Immunostaining was performed with p-Histone H3 (Ser10) primary antibody (catalog no.: 9701; CST) at room temperature for 2 h. Coverslips were then washed four times with 1× PBS and incubated at room temperature for 1 h with Alexa Fluor 568 goat anti-rabbit secondary antibody (catalog no.: A11011; Life Technologies). After incubation, the coverslips were washed four times with 1× PBS and mounted in Vectashield Vibrance antifade mounting media with 4′,6-diamidino-2-phenylindole (DAPI; catalog no.: H-1800; Vector Laboratories). Images were acquired with a Leica SP8 system confocal microscope and shown as maximum projections. For BrdU immunostaining, T80, T29, SKOV3, TOV112D, and HEY cells were first plated on glass coverslips with poly-l-lysine coating and then treated with DMSO, URMC099, or CEP1347 for 24 h. After 20 h of treatment with the appropriate inhibitor, BrdU was added to the culture medium to a final concentration of 3 μg/ml (catalog no.: 15580; Cayman Chemical); BrdU labeling was then carried out at 37 °C for 4 h in tissue culture incubator. Cells were then washed with 1× PBS, fixed with 4% paraformaldehyde, and permeabilized with 0.1% Triton X-100 as previously described. Fixed samples were washed three times with 1× PBS and treated for 30 min with 2 N HCl to allow denaturation of the DNA. Coverslips were washed three times with 1× PBS and blocked overnight with 5% BSA in 1× PBS at 4 °C. Immunostaining was performed as described previously using BrdU primary antibody (catalog no.: 32323; SCBT) and Alexa Fluor 488 goat antimouse secondary antibody (catalog no.: A11017; Life Technologies). Coverslips were mounted in Vectashield Vibrance antifade mounting media with DAPI and imaged with an Olympus IX81 inverted fluorescence microscope. Scale bars of 20 μm were added to merged images using ImageJ (National Institutes of Health).

### Flow cytometry analysis

Flow cytometry was performed to assessed cell cycle progression of T80, T29, SKOV3, TOV112D, and HEY cells treated with DMSO, URMC099, or CEP1347 for 24 h. After treatment, cells were collected by trypsinization (including any mitotic cells that were floating in the medium) followed by resuspension in 1× PBS. Resuspended cells were then fixed with 70% ethanol overnight at −20 °C. Fixed cells were then washed with cold 1× PBS and incubated on ice for 15 min with 0.25% Triton X-100 in 1× PBS. Cells were then washed with cold 1× PBS and incubated in the dark with 1× PBS containing 1% BSA and DAPI for 15 min at room temperature with gentle mixing. After incubation, cells were washed with cold 1× PBS and transferred into polystyrene round-bottom tubes for flow cytometry analysis using BD FACSDiva 8.0.1 (BD Biosciences), with 20,000 events recorded for each sample. Data were analyzed by FlowJo software (version 10.8.1; BD Biosciences).

### Quantification and statistical analysis

Quantification and statistical analyses were performed as described in the figure legend. Densitometric analyses of immunoblots were performed using ImageJ. The signal of phosphorylated protein was divided by the total protein, normalized to β-actin, and then compared with the appropriate control. The amount of shifted MLK3 was quantified by dividing the amount of shifted protein by nonshifted, normalized to β-actin, and then compared with the appropriate control. Statistical analysis was performed by two-tailed unpaired Student's *t* test on three independent biological replicates using GraphPad Prism software (version 9.3.0; GraphPad Software, Inc). For every scatter plot bar graph, bars represent mean ± SD with a *p* value ≤0.05 considered statistically significant. Asterisks were used to indicate significance: ∗*p* ≤ 0.05, ∗∗*p* ≤ 0.01, ∗∗∗*p* ≤ 0.001, and ∗∗∗∗*p* ≤ 0.0001.

## Data availability

We included all the data necessary in the interpretation of this study. Reagents and constructs are available upon request.

## Supporting information

This article contains supporting information.

## Conflict of interest

The authors declare that they have no conflicts of interest with the contents of this article.
