# Supplementary material for: Phosphorylation of mixed lineage kinase MLK3 by cyclin-dependent kinases CDK1 and CDK2 controls ovarian cancer cell division
Source: J Biol Chem. 2022 Jul 14;298(8):102263. doi: 10.1016/j.jbc.2022.102263 (PMC9399292; doi:10.1016/j.jbc.2022.102263)
Supplement: Figure S1 [file mmc1.pdf]

Fig. S1

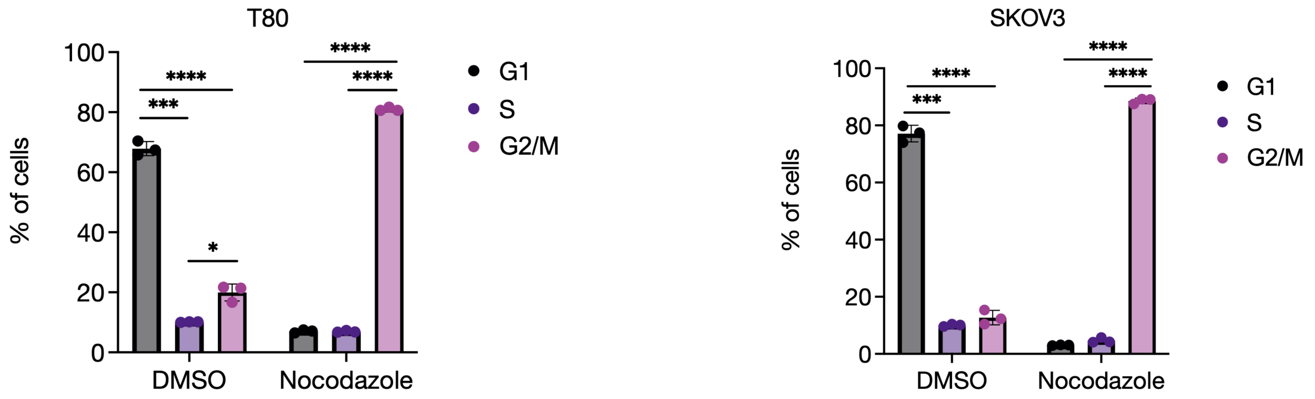

Figure S1. Cell cycle distribution of T80 and SKOV3 cells treated with nocodazole. Flow cytometry analysis of T80 and SKOV3 cells treated with DMSO or Nocodazole (1 μg/ml) for 16 h. All results represent three independent biological replicates (n=3). Results are reported as mean ± SD; *P ≤ 0.05, **P ≤ 0.01 and ****P ≤ 0.0001.
