# Supplementary material for: Phosphorylation of mixed lineage kinase MLK3 by cyclin-dependent kinases CDK1 and CDK2 controls ovarian cancer cell division
Source: J Biol Chem. 2022 Jul 14;298(8):102263. doi: 10.1016/j.jbc.2022.102263 (PMC9399292; doi:10.1016/j.jbc.2022.102263)
Supplement: Figure S2 [file mmc2.pdf]

Fig. S2

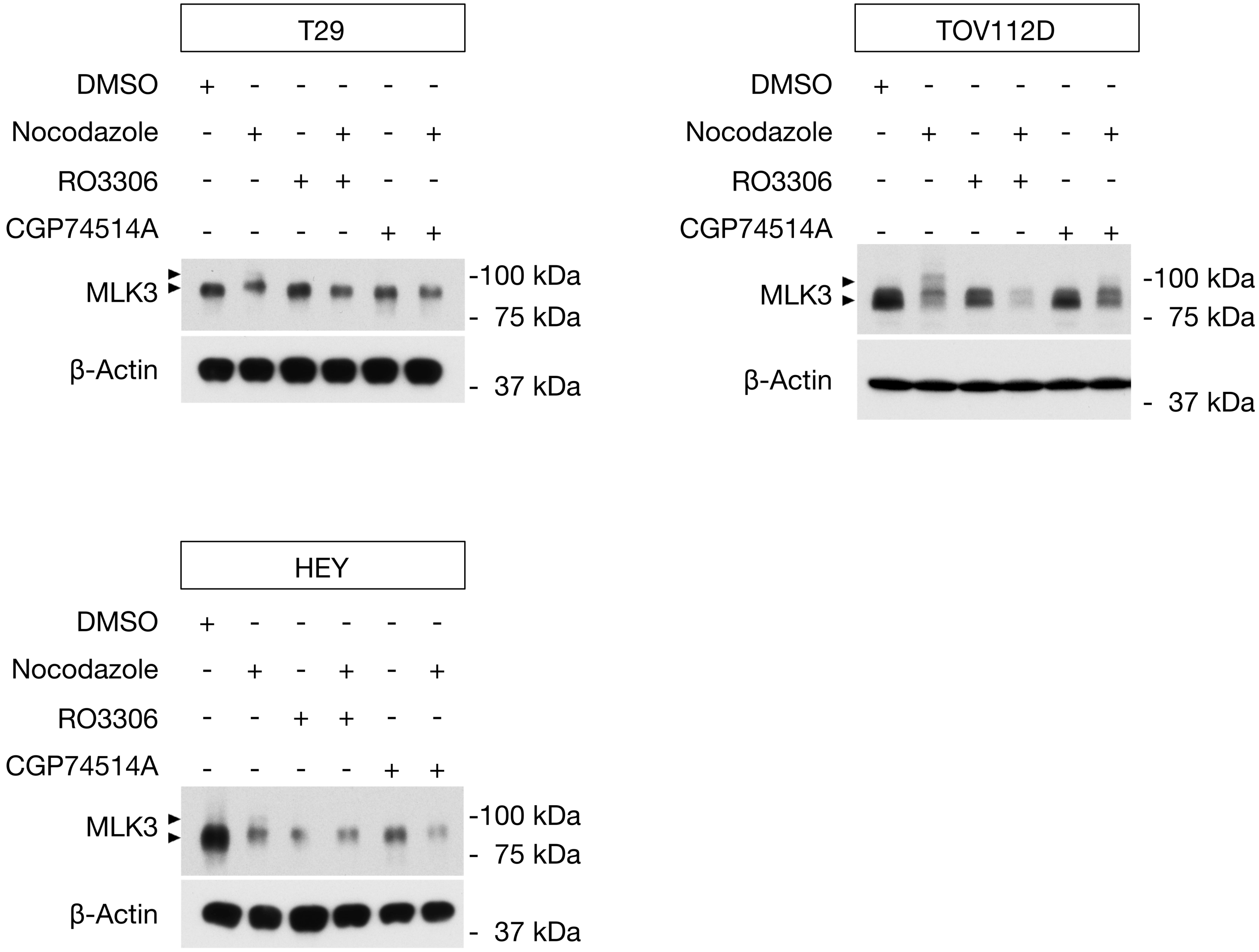

Figure S2. MLK3 phosphorylation is blocked by CDK1 inhibition in T29 normal ovarian cells and TOV112D and HEY ovarian cancer cells. T29, TOV112D and HEY cells were treated with RO3306 or CGP74514A alone or in combination with nocodazole (30 min RO3306 or CGP74514A treatment). Samples were analyzed by immunoblotting for the indicated proteins.
