# Supplementary material for: Phosphorylation of mixed lineage kinase MLK3 by cyclin-dependent kinases CDK1 and CDK2 controls ovarian cancer cell division
Source: J Biol Chem. 2022 Jul 14;298(8):102263. doi: 10.1016/j.jbc.2022.102263 (PMC9399292; doi:10.1016/j.jbc.2022.102263)
Supplement: Figure S3 [file mmc3.pdf]

Fig. S3

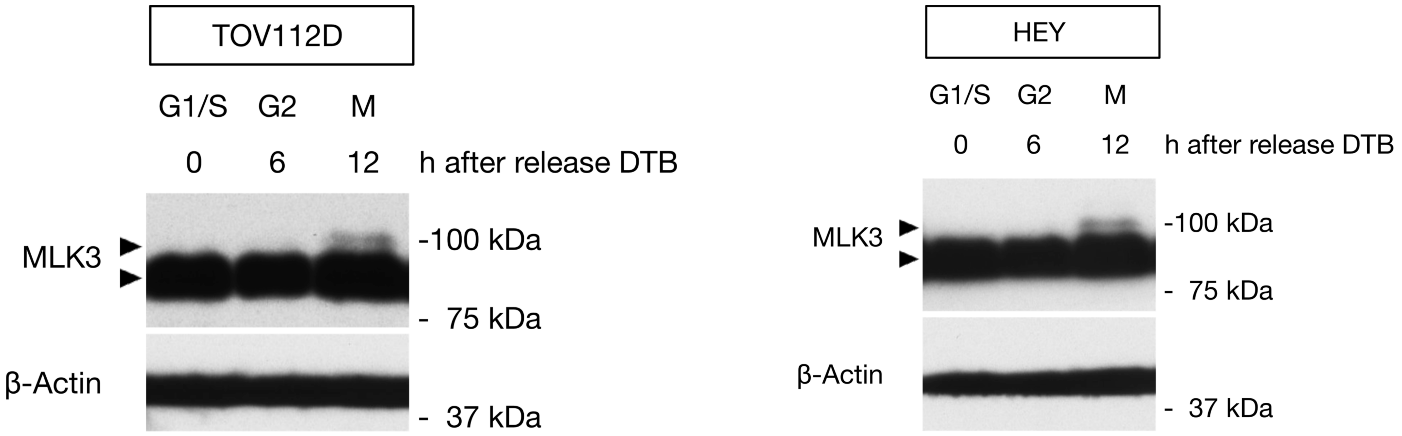

Figure S3. MLK3 is phosphorylated during mitosis in TOV112D and HEY ovarian cancer cells. TOV112D and HEY cells were synchronized by double thymidine blocked (DTB). Whole cell extracts of different time points after DTB release were analyzed by SDS PAGE and immunoblotted with the indicated antibodies.
