# Supplementary material for: Phosphorylation of mixed lineage kinase MLK3 by cyclin-dependent kinases CDK1 and CDK2 controls ovarian cancer cell division
Source: J Biol Chem. 2022 Jul 14;298(8):102263. doi: 10.1016/j.jbc.2022.102263 (PMC9399292; doi:10.1016/j.jbc.2022.102263)
Supplement: Figure S4 [file mmc4.pdf]

Fig. S4

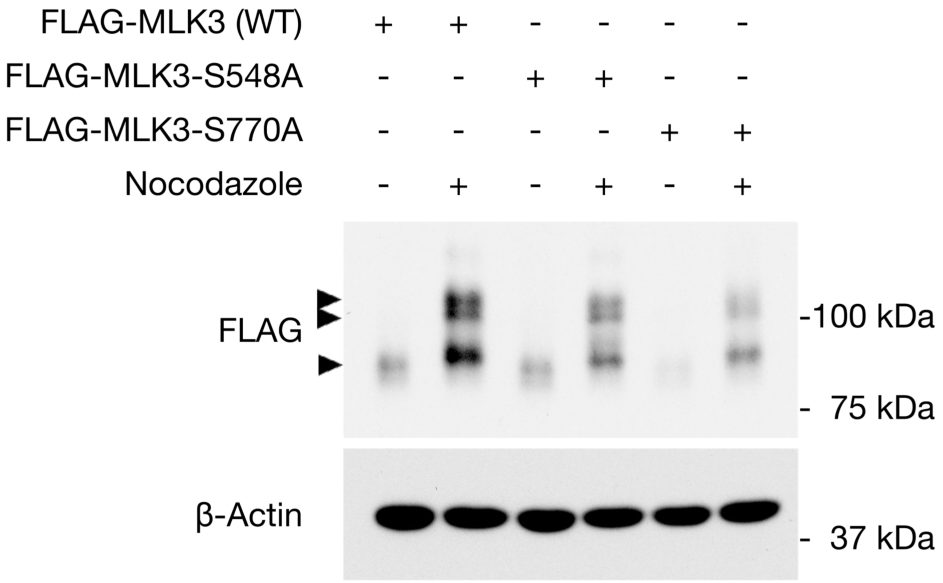

Figure S4. Reduced phosphorylation of MLK3 phospho mutants in nocodazole treated cells. FLAG-MLK3-WT, FLAG-MLK3-S548A and FLAG-MLK3-S770A were expressed in HEK293 cells. After 24 h transfection, cells were treated with DMSO or nocodazole and whole cell extracts were analyzed by immunoblotting to assessed MLK3 mobility shift.
