# Supplementary material for: Phosphorylation of mixed lineage kinase MLK3 by cyclin-dependent kinases CDK1 and CDK2 controls ovarian cancer cell division
Source: J Biol Chem. 2022 Jul 14;298(8):102263. doi: 10.1016/j.jbc.2022.102263 (PMC9399292; doi:10.1016/j.jbc.2022.102263)
Supplement: Figure S5 [file mmc5.pdf]

Fig. S5

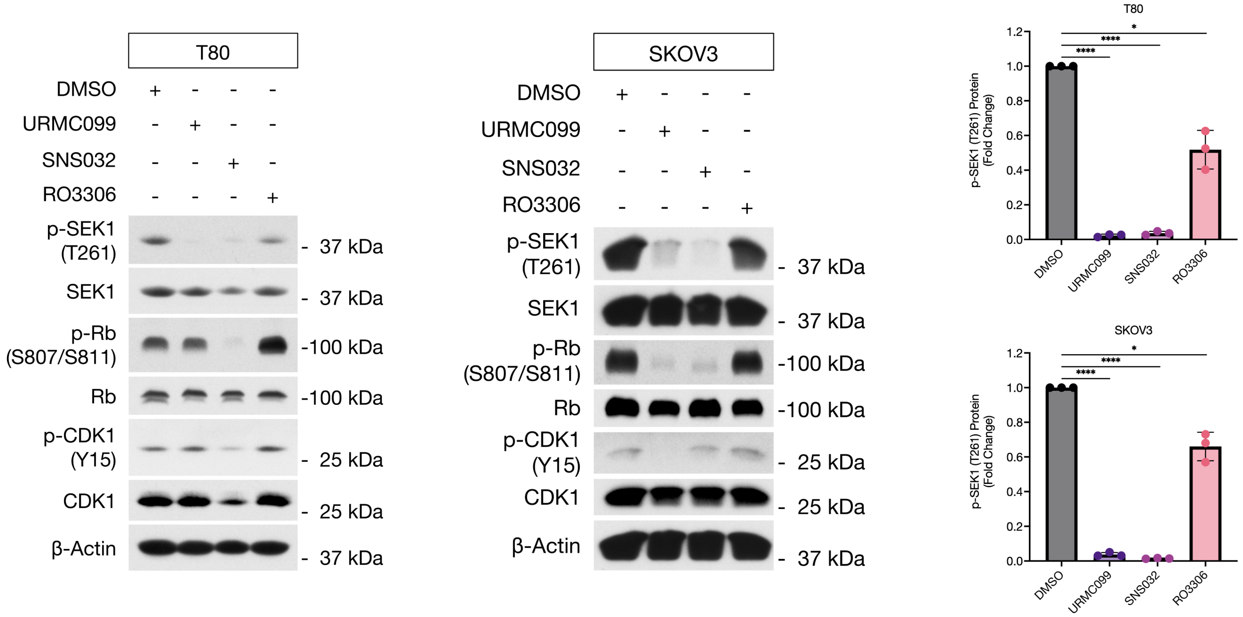

Figure S5. MLK3/SEK1 activity is reduced by CDK1 and CDK2 inhibition. T80 and SKOV3 cells treated with DMSO, URMC099, SNS032 or RO3306 for 24 h. Whole cell extracts were analyzed by SDS PAGE and immunoblotted with the indicated antibodies (n=3). All densitometric analyses represent three independent biological replicates (n=3). Results are reported as mean ± SD; *P ≤ 0.05, **P ≤ 0.01 and ****P ≤ 0.0001.
