# Supplementary material for: Phosphorylation of mixed lineage kinase MLK3 by cyclin-dependent kinases CDK1 and CDK2 controls ovarian cancer cell division
Source: J Biol Chem. 2022 Jul 14;298(8):102263. doi: 10.1016/j.jbc.2022.102263 (PMC9399292; doi:10.1016/j.jbc.2022.102263)
Supplement: Figure S6 [file mmc6.pdf]

Fig. S6

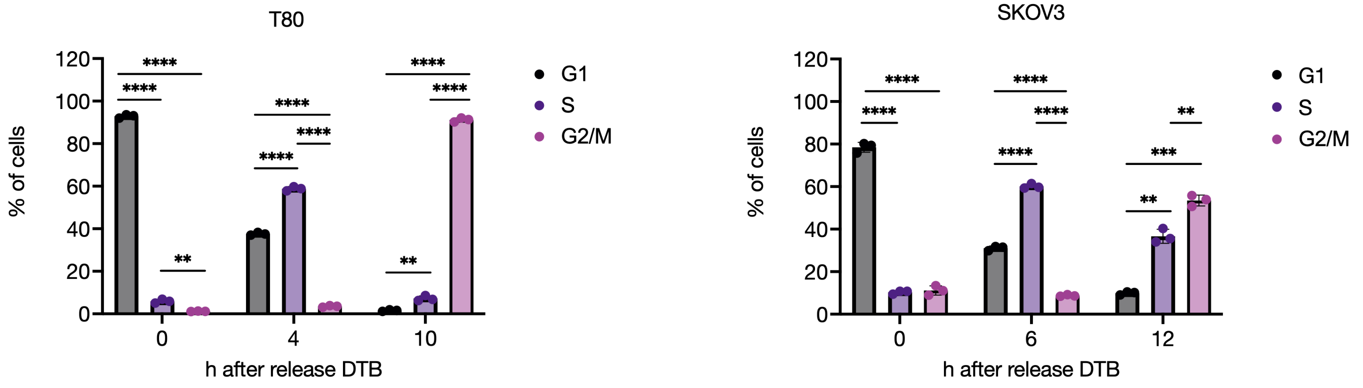

Figure S6. Cell cycle distribution of T80 and SKOV3 cells after release from double thymidine block (DTB). Flow cytometry analysis of T80 and SKOV3 cells after release from DTB. All results represent three independent biological replicates (n=3). Results are reported as mean ± SD; *P ≤ 0.05, **P ≤ 0.01 and ****P ≤ 0.0001.
